# Angioimmunoblastic T Cell Lymphoma Mimicking Chronic Urticaria

**DOI:** 10.1155/2016/8753235

**Published:** 2016-01-26

**Authors:** Mohleen Kang, Nitasha Bhatia, Adrienne Sauder, Mirela Feurdean

**Affiliations:** ^1^Department of Medicine, Rutgers New Jersey Medical School, Newark, NJ 07103, USA; ^2^Department of Pathology, Rutgers New Jersey Medical School, Newark, NJ 07103, USA

## Abstract

Angioimmunoblastic T cell lymphoma (AITL) is a rare but distinct type of T cell lymphoma with an aggressive course and high mortality. Most patients are diagnosed late in the disease and usually present with generalized lymphadenopathy. A minority have skin lesions at the time of diagnosis, more commonly in the form of nonspecific maculopapular rash with or without pruritus. We report a rare case of AITL presenting with chronic, recurrent angioedema and urticaria-like lesions and no palpable peripheral adenopathy. Primary Care physicians, dermatologists, and allergists must maintain a high index of suspicion for cutaneous manifestations of lymphoma, especially if the skin lesions are refractory to standard treatment. Timely diagnosis is essential to improve survival.

## 1. Introduction

Peripheral T cell lymphomas (PTCL) represent less than 15–20% of all non-Hodgkin's lymphomas in adults. Angioimmunoblastic T cell lymphoma (AITL) is a rare but distinct type of T cell lymphoma with an aggressive course and high mortality. It accounts for approximately 1% to 2% of non-Hodgkin's lymphoma and 15% to 20% of PTCL [1]. Most patients (89%) are diagnosed late in the disease and usually present with generalized lymphadenopathy (76%) [1]. A minority (21%) have skin lesions at the time of diagnosis, more commonly in the form of nonspecific exanthema mimicking a viral rash or a drug eruption [2]. While autoimmune paraneoplastic manifestations are common in AITL and can precede the diagnosis by many years, the association between angioedema, chronic urticaria, and T cell lymphoma is very rare [2, 3]. The index of suspicion for cutaneous manifestations of lymphoma must be high, especially if the skin lesions are refractory to standard treatment. Timely diagnosis is essential to improve survival.

## 2. Presentation

A 56-year-old Hispanic male with hypertension and two-year history of chronic idiopathic urticaria with recurrent episodes each lasting about a week presented to the emergency department (ED) with facial edema, rash, pruritus, and difficulty breathing. The patient stated that he woke up from sleep feeling his “throat was closing” and with hoarse voice. He reported an erythematous, pruritic rash over his back, chest, and abdomen that had been present for the past month. Prior to arriving at the emergency room, the patient used an epinephrine pen but noted no improvement in symptoms.

The patient was not on any antihypertensive medications and denied family history of angioedema or urticaria.

He had been diagnosed and suffering from “recurrent idiopathic angioedema and urticaria” for the past 2 years. The first episode involved right ankle edema, which progressed over the course of three weeks to the entire right body and face. The patient was admitted and treated with intravenous steroids and discharged on oral prednisone. Extensive work-up failed to find a trigger, and amlodipine was discontinued “as a precaution.” Over the next 2 years he developed multiple episodes of angioedema associated with urticaria-like lesions (erythematous, pruritic rash) lasting at least one week and sometimes longer, which resolved with steroids and antihistamines. Work-up at the time revealed monoclonal gammopathy of undetermined significance (MGUS) and normal bone marrow biopsy, skin biopsy, and flow cytometry. CT abdomen and pelvis revealed bilateral shotty inguinal lymphadenopathy, which on biopsy was noted to be reactive. He had a negative gallium scan, normal C3 and C4 levels, negative ANA, and normal CRP but elevated ESR.

About 6 months before this admission he was seen by a rheumatologist for complaints of nonspecific polyarthralgia and pruritic rash and was treated with a course of methotrexate and prednisone for presumptive seronegative rheumatoid arthritis. Work-up done at the time was notable for normal complement levels (C3, C4, and CH50), negative Hepatitis B, Hepatitis C, and HIV serologies, negative myeloperoxidase antibodies, proteinase 3 antibodies, C- or P- antineutrophil cytoplasmic antibodies, negative HLA B27 antigen, and negative rheumatoid factor, anti-citrullinated protein antibodies, and anti-SSA and anti-SSB antibodies. A skin biopsy of the rash was suggested given the “atypical appearance” but the patient did not follow up. Later, an outside allergist diagnosed him with chronic idiopathic urticaria and placed him on chronic steroid therapy.

For the month prior to admission, the patient reported increase in frequency and severity of episodes of angioedema requiring three hospital admissions; each episode lasted about a week. Contrary to the past, these episodes had minimal response to intravenous steroids. He also noted that the urticaria-like lesions had been persistent over the last month and had not improved or resolved with treatment.

**Figure 1:**
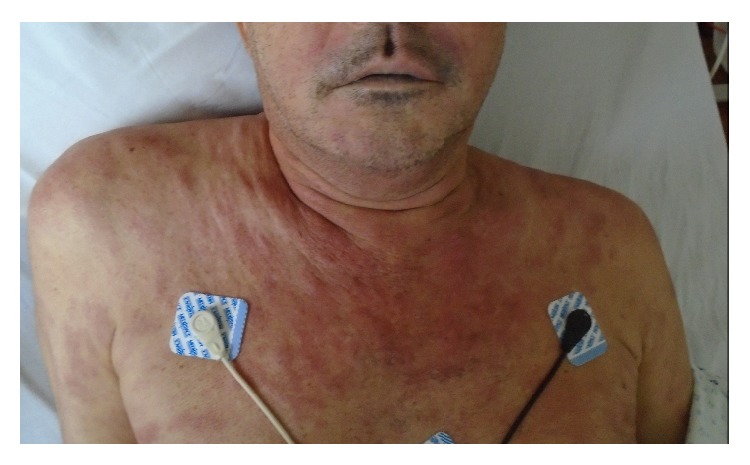
Picture of the patient taken on day of admission showing upper lip edema and diffuse, elevated, blanching, red-violaceous confluent lesions over the face, neck, and anterior chest.

**Figure 2:**
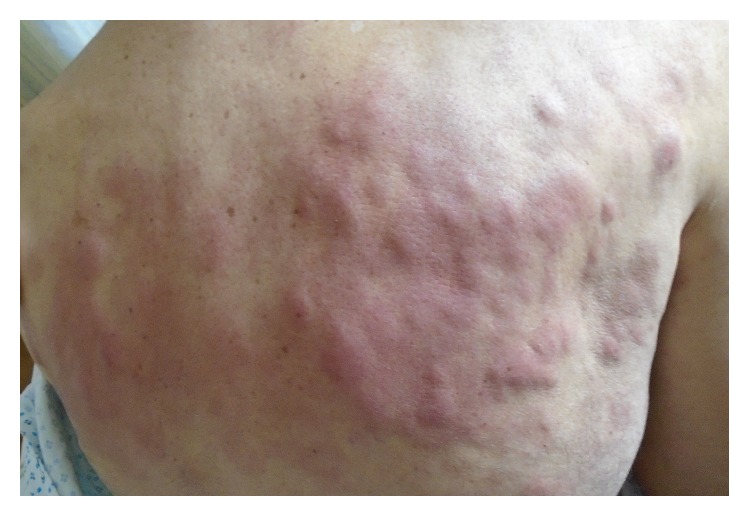
Picture of the patient taken on the day of admission showing again the diffuse elevated blanching red-violaceous confluent lesions over the back.

Physical examination revealed periorbital, upper lip edema without tongue involvement (Figure 1). Diffuse, elevated, blanching, red-violaceous confluent lesions were noted over the face, neck, and anterior and posterior chest, with a few scattered lesions on the torso and legs (Figure 2). There was no appreciable peripheral adenopathy. A bedside laryngoscopy revealed nonobstructive posterior pharyngeal edema.

Work-up done during this admission revealed the following: normal CBC, normal ANA and RF, normal complement levels (C3, C4, and CH50), normal C1Q complement qualitative and quantitative level, normal C1 esterase inhibitor level and function, normal IgE level and normal serum specific IgE for aeroallergens and for latex, negative RAST (Northeast Panel) test, negative Mycoplasma IgM antibodies, and negative anti-thyroperoxidase antibodies. Serum protein electrophoresis revealed mild hypergammaglobulinemia and monoclonal gammopathy, with normal levels of free kappa and lambda chains in the serum. Hepatitis B, Hepatitis C, HIV, and HTLV I/II serologies were negative. Quantiferon gold was indeterminate.

LDH was elevated, but AST/ALT were normal. Beta-2 microglobulin was elevated at 6.5 (normal <2.4) as was the chronic urticaria index (28.2, with normal <10). Serum tryptase level was normal (<1 ng/mL). The chest X-ray on admission was normal.

**Figure 3:**
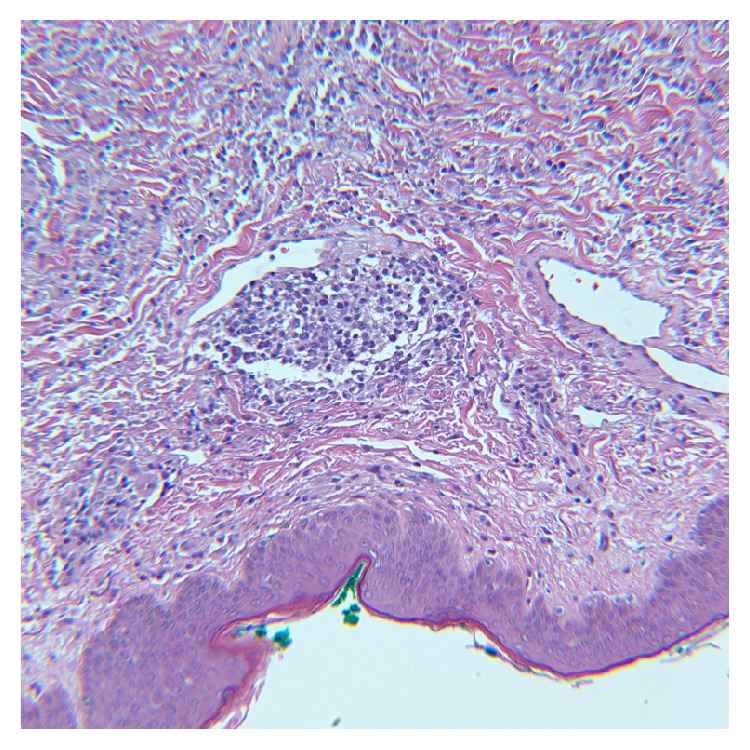
H&E section of skin biopsy showing a dense lymphohistiocytic dermal infiltrate composed predominantly of atypical lymphoid cells ranging in cell size (20x).

**Figure 4:**
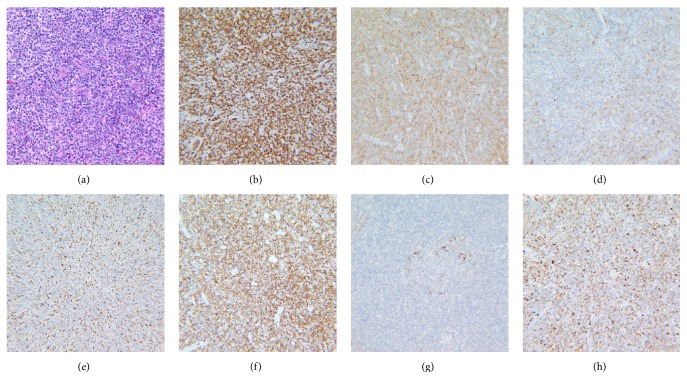
Composite: (a) H&E section of lymph node biopsy with an atypical infiltrate that is polymorphic with scattered large cells and prominent vascularity (40x). (b) Atypical lymph node cells showing immunoreactivity to CD3 (20x). (c) Atypical lymph node cells showing weak immunoreactivity to CD4 (20x). (d) Atypical lymph node cells showing partial immunoreactivity to CD10 (20x). (e) Atypical lymph node cells showing immunoreactivity to BCL-6 (20x). (f) Atypical lymph node cells showing immunoreactivity to CD5 (20x). (g) CD21 stain highlighting the follicular dendritic cells in a pattern consistent with angioimmunoblastic T cell lymphoma (20x). (h) Atypical lymph nodes cells showing immunoreactivity to perforin (20x).

The patient was admitted with a presumptive diagnosis of refractory idiopathic angioedema and chronic urticaria and was treated with intravenous corticosteroids, H1 + H2 antihistamines, and mycophenolate mofetil, but the angioedema continued to worsen. Given the severity of the symptoms and lack of improvement, he was given a trial of two doses of Icatibant without any response. A CT Scan of the neck, chest, and abdomen obtained nine days after admission revealed diffuse lymphadenopathy (cervical, mediastinal, retroperitoneal, and pelvic) and no hepatosplenomegaly. A biopsy of the skin lesions on the posterior chest and an excisional cervical lymph node biopsy revealed angioimmunoblastic T cell lymphoma (AITL) with cutaneous involvement (Figures 3 and 4). Later, the patient went into respiratory failure. Given his deteriorating clinical status at the time of diagnosis, he was not a candidate for chemotherapy. Sadly, he died within a week from sepsis and multiorgan failure.

## 3. Discussion

Angioimmunoblastic T cell lymphoma is a rare but distinct type of T cell lymphomas with aggressive course and high mortality. It accounts for approximately 1% to 2% of non-Hodgkin's lymphoma, with an annual incidence of 0.05 new cases per 100,000 people in US. Most patients are diagnosed late in the disease (89%) and usually present with generalized lymphadenopathy, hepatosplenomegaly, and constitutional symptoms of fever and weight loss. Involvement of more than one extranodal site and elevation of beta-2 microglobulin portend poor prognosis [2, 4, 5].

Although pathogenesis of AITL remains unknown, it has been associated with a number of infectious diseases including tuberculosis, Cryptococcus neoformans, and lymphotropic viruses such as EBV, HHV 6, HHV 8, HIV, and Hepatitis C [3]. The best evidence to date supports EBV infection in the pathogenesis of AITL, as EBV infected cells have been found in up to 95% of AITL cases [3, 6, 7]. In our patient EBV in situ hybridization was positive. The pathogenetic mechanism remains hypothetical and includes virus induced immunosuppression and deregulated cellular signaling [3].

Autoimmune phenomena described in association with AITL include the presence of circulating immune complexes, cold agglutinins, autoimmune hemolytic anemia, positive anti-smooth-muscle antibodies and rheumatoid factor, and autoimmune thyroid disease [3]. There is no clear pathogenetic model that links malignant lymphoid transformation with the development of autoimmunity; loss of adaptive immunity through malignant transformation of B and T cells has been proposed [8]. Hypergammaglobulinemia is present in 30–50% of patients and is typically polyclonal; however, up to 10% of patients may present with monoclonal gammopathy [1]. Seropositive rheumatoid arthritis has been described as well [3]. In hindsight, our patient's polyarticular pains and the persistent MGUS were likely paraneoplastic manifestations of AITL, which preceded by months to years the histopathologic diagnosis.

AITL is associated with frequent extranodal and paraneoplastic manifestations (27%). Cutaneous manifestations of AITL are rare and usually present as nonspecific maculopapular rash which resembles a viral exanthema, or a drug reaction [1, 2]. Pruritus can be a prominent symptom, as it was in our patient. Angioedema as the initial presenting symptom of AITL is exceedingly rare but has been reported before [9, 10]. The association of angioedema and chronic urticaria with AITL is even more unusual.

In our patient, the complement levels were normal, as were C1 esterase level and functional assays that are often abnormal in hereditary angioedema types I and II and in acquired angioedema; hence these diagnoses were virtually excluded [11, 12].

Chronic urticaria is usually classified as either inducible or spontaneous. Chronic spontaneous urticaria is defined as urticaria without any identifiable cause, lasting more than 6 weeks, with or without angioedema. H1 antihistamines are the main stay of treatment. Wheals are usually pruritic and fleeting and resolve within 24 hours, while angioedema can persist up to 72 hours [13]. Our patient's rash was pruritic but it did not resolve within 24 hours and was refractory to treatment with antihistamines, which prompted the rheumatologist to suggest a biopsy months before the admission which led to the final diagnosis (see Table 1).

The Chronic Urticaria (CU) Index is an in vitro assay that measures the histamine levels after a patient's serum is mixed with donor basophils. An elevated CU Index (greater than or equal to 10) indicates either an autoimmune cause for urticaria or the presence of an alternate histamine releasing factor [14, 15]. While the CU index was elevated in our patient, it may have been so due to histamine releasing factors secreted locally by tumor cells and not necessarily due to autoimmune causes such as antibodies against IgE, Fc*ε*RI, or anti-Fc*ε*RII.

Given the lack of response to antihistamines, steroids, mycophenolate (suppressor of T-lymphocytic and primary antibody responses to antigens), Icatibant (selective, bradykinin B2 receptor antagonist), and fresh frozen plasma (replacement of defective C1 esterase inhibitor), it is likely that the acquired angioedema and urticaria-like lesions occurred without mastocyte degranulation and systemic histamine release, activation of the complement cascade, or excessive endogenous bradykinin production, suggesting local release of vasoactive molecules by the T cells infiltrating the skin.

Moreover, presence of lymphadenopathy in the setting of complement and/or bradykinin mediated angioedema is unusual and should prompt further work up to rule out malignancy. It is unclear whether our patient underwent an excisional biopsy in Puerto Rico the year before and if flow cytometry was performed on the sample; fine needle or core needle biopsies without flow cytometry make diagnosis of lymphomas difficult [16].

In conclusion, recurrent episodes of angioedema lasting for more than 3–5 days in the setting of an atypical urticaria which does not resolve with treatment within 24 hours should prompt further investigation. Presence of lymphadenopathy in the setting of angioedema is often a hallmark of underlying malignancy and should be investigated with a thorough workup until it is ruled out.

## Figures and Tables

**Table 1:** Typical features of chronic spontaneous urticaria versus features in our patient.

Features	Chronic spontaneous urticaria	Our patient
Clinical appearance	Sudden onset of wheals, angioedema, or both	Sudden onset of angioedema and wheals

Duration of symptoms	Recurrent episodes for >6 weeks	Recurrent episodes for almost 2 years
	Wheals resolve within 24 hours Angioedema resolves within 72 hours	Episodes of angioedema and wheals lasting more than one week

Triggers	Not identifiable	Not identifiable

Presence of lymphadenopathy	No	Yes

Response to treatment	Most cases respond to H1 antihistamines	Initially responded to H1 antihistamines and steroids, in time became refractory to therapy

## References

[1] Federico M., Rudiger T., Bellei M. (2013). Clinicopathologic characteristics of angioimmunoblastic T-cell lymphoma: analysis of the international peripheral T-cell lymphoma project. *Journal of Clinical Oncology*.

[2] Ferran M., Gallardo F., Baena V., Ferrer A., Florensa L., Pujol R. M. (2006). The ‘deck chair sign’ in specific cutaneous involvement by angioimmunoblastic T cell lymphoma. *Dermatology*.

[3] Dogan A., Attygalle A. D., Kyriakou C. (2003). Angioimmunoblastic T-cell lymphoma. *British Journal of Haematology*.

[4] Yoo C., Yoon D. H., Suh C. (2014). Serum beta-2 microglobulin in malignant lymphomas: an old but powerful prognostic factor. *Blood Research*.

[5] Yun G. J., Kim K. M., Bae Y.-J. (2010). Cutaneous NK/T-cell lymphoma preceded by persistent facial angioedema. *Acta Dermato-Venereologica*.

[6] Balaraman B., Conley J. A., Sheinbein D. M. (2011). Evaluation of cutaneous angioimmunoblastic T-cell lymphoma. *Journal of the American Academy of Dermatology*.

[7] Ocampo-Garza J., Herz-Ruelas M. E., González-Lopez E. E. (2014). Angioimmunoblastic T-cell lymphoma: a diagnostic challenge. *Case Reports in Dermatology*.

[8] Stern M., Buser A. S., Lohri A., Tichelli A., Nissen-Druey C. (2007). Autoimmunity and malignancy in hematology—more than an association. *Critical Reviews in Oncology/Hematology*.

[9] Harrison N. K., Twelves C., Addis B. J., Newman Taylor A. J., Souhami R. L., Isaacson P. G. (1988). Peripheral T-cell lymphoma presenting with angioedema and diffuse pulmonary infiltrates. *The American Review of Respiratory Disease*.

[10] Chen L. Y. C., Lai E. J., Collins D. R., Ostrow D. N., Sreenivasan G. M. (2010). A young woman with episodic angioedema, papilledema, and eosinophilia. *American Journal of Hematology*.

[11] Bernstein J. A., Moellman J. (2012). Emerging concepts in the diagnosis and treatment of patients with undifferentiated angioedema. *International Journal of Emergency Medicine*.

[12] Walford H. H., Zuraw B. L. (2014). Current update on cellular and molecular mechanisms of hereditary angioedema. *Annals of Allergy, Asthma & Immunology*.

[13] Zuberbier T., Aberer W., Asero R. (2014). The EAACI/GA^2^LEN/EDF/WAO Guideline for the definition, classification, diagnosis, and management of urticaria: the 2013 revision and update. *Allergy*.

[14] Altrich M. L., Halsey J. F., Altman L. C. (2009). Comparison of the in vivo autologous skin test with in vitro diagnostic tests for diagnosis of chronic autoimmune urticaria. *Allergy and Asthma Proceedings*.

[15] Biagtan M. J., Viswanathan R. K., Evans M. D., Mathur S. K. (2011). Clinical utility of the Chronic Urticaria Index. *Journal of Allergy and Clinical Immunology*.

[16] Zeppa P., Vigliar E., Cozzolino I. (2010). Fine needle aspiration cytology and flow cytometry immunophenotyping of non-Hodgkin lymphoma: can we do better?. *Cytopathology*.

